# Electrochemical Nanosensor for the Simultaneous Determination of Anticancer Drugs Epirubicin and Topotecan Using UiO-66-NH_2_/GO Nanocomposite Modified Electrode

**DOI:** 10.3390/bios14050229

**Published:** 2024-05-04

**Authors:** Somayeh Tajik, Parisa Shams, Hadi Beitollahi, Fariba Garkani Nejad

**Affiliations:** 1 Research Center of Tropical and Infectious Diseases, Kerman University of Medical Sciences, Kerman 76169-13555, Iran; 2 Department of Anatomical Sciences, Afzalipour School of Medicine, Kerman University of Medical Sciences, Kerman 76169-13555, Iran; parisashams12@gmail.com; 3 Environment Department, Institute of Science and High Technology and Environmental Sciences, Graduate University of Advanced Technology, Kerman 76318-85356, Iran; h.beitollahi@yahoo.com (H.B.); f.garkani95@gmail.com (F.G.N.)

**Keywords:** epirubicin, topotecan, modified electrode, UiO-66-NH_2_/GO nanocomposite, metal organic framework

## Abstract

In this work, UiO-66-NH_2_/GO nanocomposite was prepared using a simple solvothermal technique, and its structure and morphology were characterized using field emission scanning electron microscopy (FE-SEM), energy-dispersive X-ray spectroscopy (EDS), and X-ray diffraction (XRD). An enhanced electrochemical sensor for the detection of epirubicin (EP) was proposed, which utilized a UiO-66-NH_2_/GO nanocomposite-modified screen-printed graphite electrode (UiO-66-NH_2_/GO/SPGE). The prepared UiO-66-NH_2_/GO nanocomposite improved the electrochemical performance of the SPGE towards the redox reaction of EP. Under optimized experimental conditions, this sensor demonstrates a remarkable limit of detection (LOD) of 0.003 µM and a linear dynamic range from 0.008 to 200.0 µM, providing a highly capable platform for sensing EP. Furthermore, the simultaneous electro-catalytic oxidation of EP and topotecan (TP) was investigated at the UiO-66-NH_2_/GO/SPGE surface utilizing differential pulse voltammetry (DPV). DPV measurements revealed the presence of two distinct oxidation peaks of EP and TP, with a peak potential separation of 200 mV. Finally, the UiO-66-NH_2_/GO/SPGE sensor was successfully utilized for the quantitative analysis of EP and TP in pharmaceutical injection, yielding highly satisfactory results.

## 1. Introduction

Cancer, a grave illness, has been identified as a significant contributor to human mortality for many years. Scientists have made numerous endeavors to combat cancer. Chemotherapy stands as the primary approach in cancer treatment, relying on the utilization of anti-cancer drugs [1,2]. This treatment method can be associated with various side effects. Therefore, providing fast, accurate, and sensitive analytical methods to determine chemotherapy drugs in different samples can significantly contribute to effective treatment and reducing side effects. Epirubicin (EP) and topotecan (TP) are widely used as fundamental anti-cancer drugs in current medical practice [3]. EP, a type of anthracycline antibiotic, demonstrates remarkable efficacy as an antineoplastic drug. It finds extensive application in the treatment of diverse cancer types, such as breast cancer, gastric cancer, hepatocellular carcinoma, leukemia, and lymphoma. The mechanism of action of EP involves inhibiting DNA transcription and replication. The administration of a high dose of EP through long-term usage in cancer therapy exhibits a potent cytotoxic effect on both cancerous and healthy cells [4,5,6]. Topotecan (TP) is a semi-synthetic camptothecin derivative that is dissolved in water. It has been widely employed in the treatment of specific cancer types, including lung, breast, vaginal opening, and ovarian cancer. TP functions as an inhibitor of topoisomerase I, an essential enzyme in DNA replication that is vital for cell survival. By targeting this enzyme, TP induces cell death. When using TP during chemotherapy, major side effects such as vomiting, weakness, hair loss, and certain gastrointestinal, blood, and lymph system disorders may be observed [7,8,9]. Monitoring anticancer drugs during chemotherapy is of great importance as an overdose of these medications can have severe detrimental effects on the patients. According to scientific reports, combining EP and TP is considered an acceptable strategy for treating chemosensitive diseases.

Various measurement strategies have been employed for two primary purposes: the need to monitor chemotherapeutic medications and the necessity to control their dosage in biological specimens [10]. Electrochemical methods have become increasingly popular in the analysis of biological and pharmaceutical compounds, replacing other techniques like chromatography [11,12,13,14]. The electrochemical detection methods are commonly used for both quantitative and qualitative analysis due to their advantages over other analytical methods. These advantages include faster response times, accurate results, cost-effectiveness, and ease and efficient data collection processes [15,16,17,18,19,20].

From the diverse working electrodes accessible, the SPGEs have been chosen in our study due to their cost-effectiveness, wide availability, miniaturized morphology, fast response, and compatibility with portable devices [21,22]. One of the practical benefits of using SPEs is that they incorporate reference, auxiliary, and working electrodes into a single tool, which enhances convenience and efficiency. The SPEs have gained significant interest in recent years because they can be tailored and modified to make them suitable for the requirements of the end user, showing good potential for various commercial applications [23,24,25].

Generally, unmodified electrodes exhibit high over-potential and low sensitivity, which ultimately results in the accumulation of surface fouling over time. The modification of the electrode surface plays a crucial role in the electrochemical detection of different analytes. The primary objective of electrode modification is to enhance the exchange of electrons between the surface of the electrode and the electro-active species [26,27,28,29,30]. Therefore, numerous works have been undertaken to create modified electrodes utilizing various materials and nanostructures [31,32]. Nanomaterials offer benefits such as a high specific surface area, excellent conductivity, numerous surface-active sites, and powerful catalytic performance. These qualities can significantly enhance the sensor’s stability and sensitivity. Additionally, they can serve as catalysts in electrochemical reactions to facilitate reactions and improve the effectiveness of electron transfer [33,34,35,36].

As nanomaterials progressed, functional materials were used to modify electrodes and increase the sensitivity of detection. Metal-organic frameworks (MOFs) have demonstrated significant potential in various fields due to their extensive specific surface areas and high porosity [37,38,39,40]. UiO-66-NH_2_ is especially noteworthy for a variety of reasons. The original UiO-66 prototype, developed by Lillerud and his team in 2008, is the initial crystalline framework according to zirconium clusters [41]. These MOFs are commonly utilized because of their distinct characteristics, such as their ability to withstand high temperatures, chemical stability, mechanical strain, and ease of regeneration, as well as their chemical and thermal stability. The UiO-66 structure is characterized by robust connections between non-mineral blocks and organic linkers, a high coordination number, and sturdy O-Zr bonds. Their ability to be adjusted and adapted in both structure and function makes them attractive options for a wide range of uses, such as heterogeneous catalysis, gas adsorption, energy devices, sensing, and medical applications [42,43,44,45,46]. Recent studies indicated that the combination of MOFs with other nanostructures can not only enhance their electrical conductivity but also significantly improve the stability and electro-catalytic ability of modified electrodes [47,48,49].

Carbon nanostructures, including graphene oxide (GO) and its derivatives, carbon nanotubes, fullerene, mesoporous carbon, etc., are materials that are very different in structure and properties and include diverse and wide applications [50,51]. Graphene is one of the allotropes of carbon, and with its discovery, great achievements have been made in the field of carbon nanostructures. Single-layer graphene sheets are formed by arranging carbon atoms together in a two-dimensional honeycomb configuration. Despite the many features of GO, features such as its high surface area and electrical conductivity led to GO being used as a suitable and practical material in many fields of electrochemistry, including electrochemical sensing, energy storage and conversion, etc. [52,53,54,55,56].

In this study, we have designed an electrochemical sensing platform by combining SPGE and UiO-66-NH_2_/GO nanocomposite for the detection of EP. The SPGE surface was coated with a suspension of UiO-66-NH_2_/GO nanocomposite, which served as a platform to enhance the signal. Cyclic voltammetry (CV), chronoamperometry, and DPV techniques were employed for electrochemical analysis. Moreover, the proposed sensor was effectively utilized for the simultaneous detection of EP and TP. In conclusion, the practicality of the developed sensor was illustrated by its successful analytical application in EP and TP injection samples.

## 2. Experimental

### 2.1. Reagents

All chemicals used in this study were of the highest available analytical grade and were obtained from Merck (Darmstadt, Germany) and Sigma-Aldrich (St. Louis, MO, USA) companies without requiring any additional purification.

### 2.2. Synthesis of UiO-66-NH_2_/GO Nanocomposite

At first, for preparation of precursors, 0.0467 g of ZrCl_4_ and 0.0363 g of 2-amino terephthalic acid in 20 mL of dimethylformamide (DMF) were dissolved by stirring for 10 min. Then, 5.0 mL of acetic acid was added dropwise to the above solution under stirring. After that, 0.0075 g of GO was added and subjected to ultrasonic waves for 45 min. The prepared suspension of GO and MOF precursors was transferred into a Teflon-lined autoclave, and it was kept at a constant temperature of 120 °C for 48 h in an oven for a solvothermal reaction. After 48 h, the autoclave cooled to room temperature naturally. The prepared precipitate was collected, washed several times with DMF and ethanol, and finally dried at 70 °C for 16 h under vacuum.

### 2.3. Preparation of Working Electrode

The modification of the SPGE surface was carried out by dropping 3.0 µL of the UiO-66-NH_2_/GO suspension (1 mg/mL in H_2_O) over the working electrode and allowing the solvent to completely evaporate at room temperature.

The electrochemically active surface areas (EASAs) were calculated by studying the redox (oxidation-reduction) reaction of the redox probe (Fe(CN)_6_^3−/4−^) at various scan rates by using CV on the bare SPGE, UiO-66-NH_2_ MOF/SPGE, GO/SPGE and UiO-66-NH_2_/GO/SPGE. The Randles-Sevcik equation (Equation (1)) was used to calculate the EASAs of electrodes.

(Ip = 2.69 × 10^5^n^3/2^AC_0_D^1/2^ʋ^1/2^) (1)

In this equation:
Ip = Current intensity
n = Number of participated electrons in the redox reaction (n = 1)
A = EASA
C_0_ = Concentration of redox probe
D = Diffusion coefficient
ʋ = Scan rate

The EASAs of the bare SPGE, UiO-66-NH_2_ MOF/SPGE, GO/SPGE and UiO-66-NH_2_/GO/SPGE was calculated to be 0.035, 0.07, 0.085 and 0.122 cm^2^, respectively.

### 2.4. Electrochemical Analysis

The electrochemical tests were conducted using a potentiostat-galvanostat Autolab PGSTAT 302N (Metrohm; The Netherlands), which was equipped with GPES 4.9 software. The potentiostat-galvanostat was connected to a SPGE ((DRP-110) (The SPGE consists of a graphite working electrode (WE), an Ag pseudo-reference electrode (RE), and a graphite counter electrode (CE)); DropSens, DRP-110, Asturias, Spain) through a dedicated cable connector, and a personal computer system was utilized for the electrochemical measurements.

All experiments were conducted in phosphate buffer solutions (PBSs) containing 0.1 M of phosphoric acid, and sodium hydroxide solution was added to adjust the pH value to the desired level. pH measurements were conducted by using a metrohm 713 pH meter (Switzerland). The CV studies were performed to compare the electrochemical behavior of EP at the unmodified and modified SPGE in phosphate buffer 0.1 M at pH 7.0 in the specified potential range (0.16–0.88 V) vs. Ag-pseudo reference electrode and scan rate of 50 mV/s. To investigate the effect of scan rate, the CV studies were carried out in 0.1 M phosphate buffer at pH 7.0 in the potential range (0.16–0.88 V) vs. Ag-pseudo reference electrode at different scan rates. To perform the chronoamperometric measurements in 0.1 M phosphate buffer at pH 7.0, a constant step-potential (670 mV) vs. Ag-pseudo reference electrode was applied to the working electrode over a certain range of time. The DPV measurements of EP were conducted in 0.1 M phosphate buffer at pH 7.0 in a potential window ranging from 0.4 V to 0.85 V. The DPV parameters were set as follows: step potential = 0.01 V and pulse amplitude = 0.025 V.

## 3. Results and Discussion

### 3.1. Characterization of UiO-66-NH_2_/GO Nanocomposite

**Figure 1:**
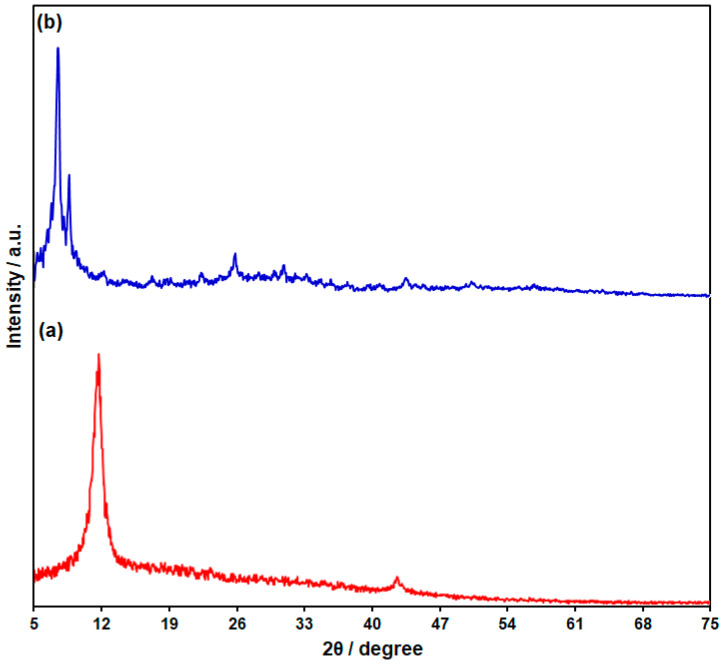
XRD patterns of GO (**a**) and UiO-66-NH_2_ MOF/GO nanocomposite (**b**).

The XRD analysis of GO and UiO-66-NH_2_ MOF/GO nanocomposite was carried out to determine the crystalline nature of samples, and their diffraction patterns are shown in Figure 1. The XRD pattern of GO reveals two peaks at 11.5° and 42.5°, which are related to the diffraction from (001) and (101) planes of graphite structure, respectively. From the XRD pattern of the nanocomposite, the observed values of 2θ for UiO-66-NH_2_ MOF were in good agreement with the diffraction patterns that were reported in previous works [57,58]. Also, the peaks of GO in the XRD pattern of nanocomposite probably overlapped with the peaks of UiO-66-NH_2_ MOF.

**Figure 2:**
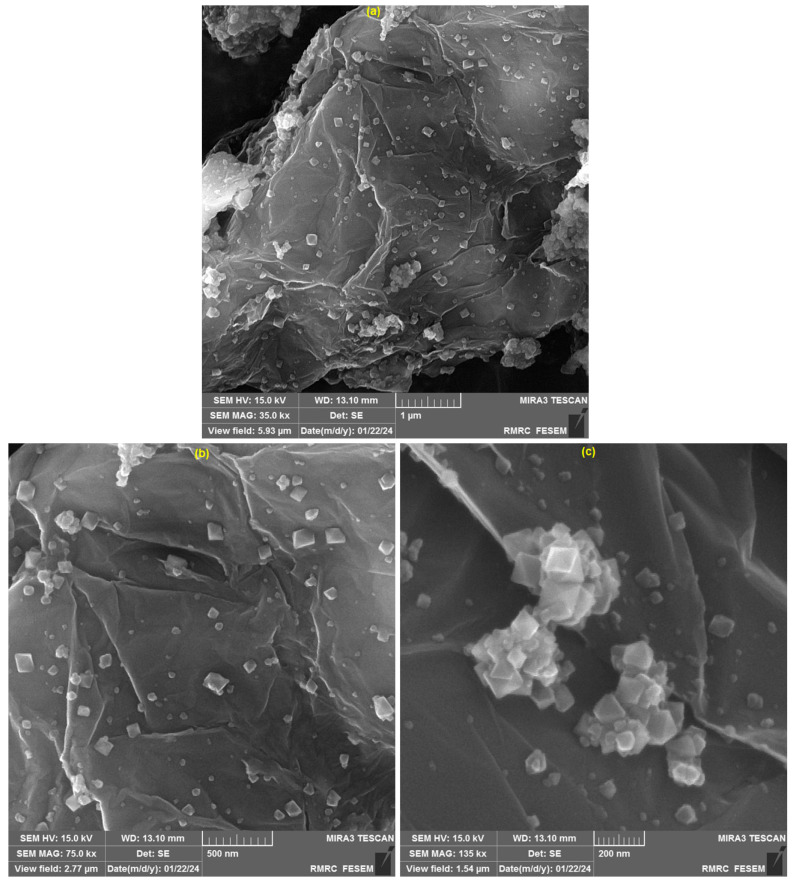
FE-SEM images of UiO-66-NH_2_ MOF/GO nanocomposite at (**a**) 1 µm, (**b**) 500 nm, and (**c**) 200 nm magnifications.

Figure 2 shows the typical morphologies of as-prepared UiO-66-NH_2_ MOF/GO nanocomposite at 1 µm magnification (a), 500 nm magnification (b), and 200 nm magnification (c) as provided by FE-SEM. From Figure 2, it is clearly that the UiO-66-NH_2_ MOF exhibits octahedral morphology with particle size of approximately 100 nm, which are formed on GO sheets.

**Figure 3:**
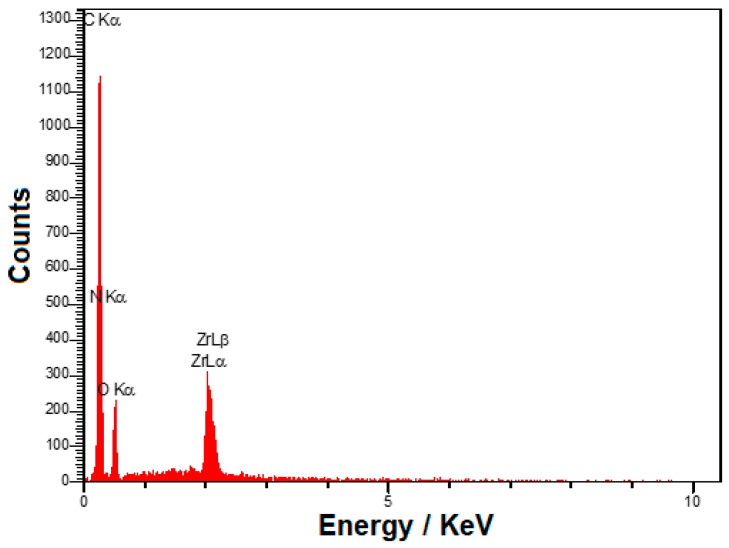
EDS spectrum of UiO-66-NH_2_ MOF/GO nanocomposite.

Furthermore, the EDS analysis for UiO-66-NH_2_ MOF/GO nanocomposite was performed to show the presence of elements including Zr, C, O, and N (Figure 3).

### 3.2. Electrochemical Behavior of EP on the Surface of UiO-66-NH_2_/GO/SPGE

**Scheme 1:**
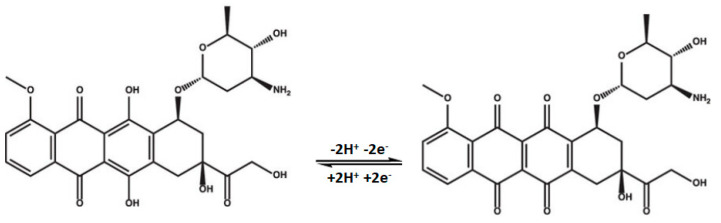
The mechanism for the redox reaction of EP.

The pH value of the buffer solution is a significant factor that plays a critical role in the electrochemical reactions. The impact of pH on determination of EP was examined in experiments using 0.1 M PBS at various pHs (2.0–9.0) containing EP. The pH 7.0 was selected as it exhibited the highest anodic peak current in DPV measurements for EP. The mechanism for the redox reaction of EP is depicted in Scheme 1.

**Figure 4:**
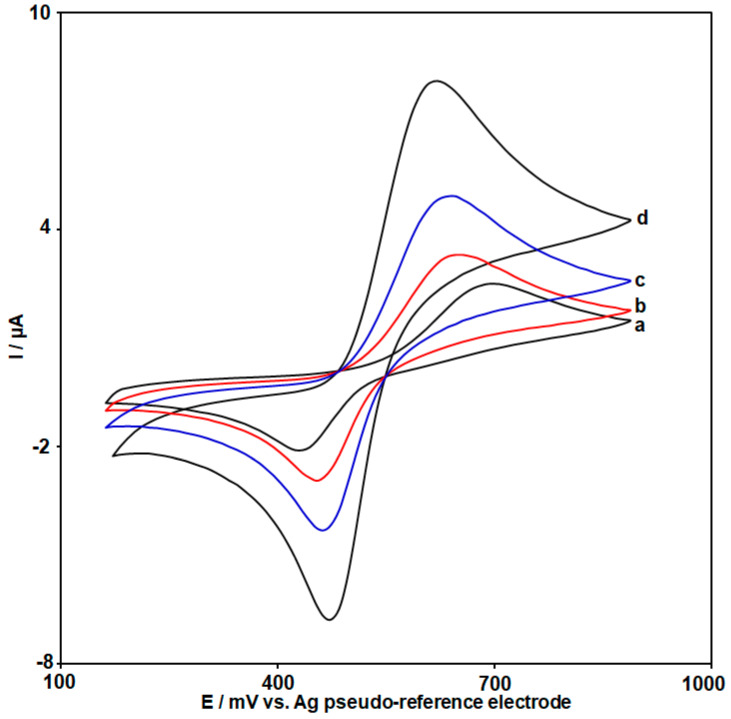
CVs obtained at bare SPGE (voltammogram a), UiO-66-NH_2_ MOF/SPGE (voltammogram b), GO/SPGE (voltammogram c), and UiO-66-NH_2_/GO/SPGE (voltammogram d) in the presence of 80.0 µM EP containing 0.1 M PBS (pH = 7.0) with the scan rate of 50 mV/s.

Figure 4 demonstrates the CVs of 80.0 µM EP in 0.1 M PBS (pH = 7.0) obtained at various electrodes (bare SPGE (cyclic voltammogram a), UiO-66-NH_2_ MOF/SPGE (cyclic voltammogram b), GO/SPGE (cyclic voltammogram c), and UiO-66-NH_2_/GO/SPGE (cyclic voltammogram d)). In the case of the bare SPGE (voltammogram a), a weak redox peak was observed for EP, which indicates the slow speed of electron transfer on the bare electrode. According to the obtained voltammogram b and voltammogram c, it is clear that the CV responses after modification of SPGE with UiO-66-NH_2_ MOF and GO towards EP determination were enhanced compared to bare SPGE. The redox peaks were observed at lower potentials with higher current values, which shows the significant role of UiO-66-NH_2_ MOF and GO in improving the performance of SPGEs. In addition, the modification of SPGE with both UiO-66-NH_2_ MOF and GO (voltammogram d) yielded better CV response towards EP than other electrodes. In this case, the highest sensitivity was obtained, and the potential of redox peaks shifted toward more negative values. The observation of these effects is a clear indication of the synergistic effect that arises from the simultaneous use of GO and UiO-66-NH_2_ MOF. In fact, the modification of SPGE with UiO-66-NH_2_ MOF and GO significantly enlarged the specific surface area, enhanced the conductivity, and improved the electron transfer ability of the electrode, which provided an excellent sensing platform for the redox reaction of EP.

### 3.3. Effect of Scan Rate

**Figure 5:**
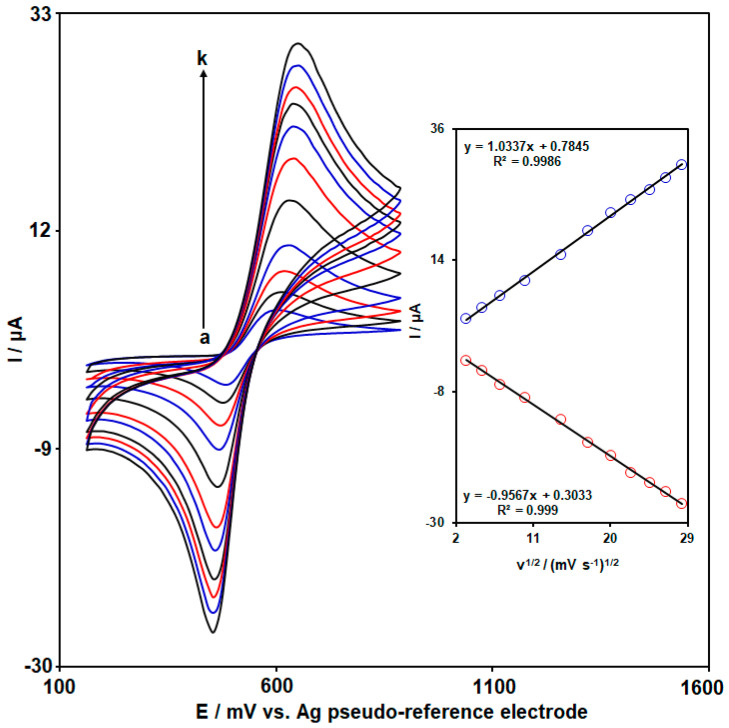
CVs of 80.0 µM EP at various scan rates (from a to k: 10 mV/s, 25 mV/s, 50 mV/s, 100 mV/s, 200 mV/s, 300 mV/s, 400 mV/s, 500 mV/s, 600 mV/s, 700 mV/s, and 800 mV/s) in 0.1 M PBS (pH = 7.0) using UiO-66-NH_2_/SPGE. Inset: the plot of Ipa and Ipc vs. ʋ^1/2^ for EP.

The influence of scan rate on the redox reaction of EP (80.0 µM) at UiO-66-NH_2_/GO/SPGE was investigated using CV (Figure 5). The peak potentials for redox peaks of EP slightly shifted towards more positive values as the scan rate increased, indicating kinetic limitations in the redox reaction of EP. Also, the anodic peak currents (Ipa) and cathodic peak currents (Ipc) of EP exhibited a linear relationship with the square root of the scan rate (ʋ^1/2^) (Figure 5-Inset). These relationships were observed over a range of 10 mV/s to 800 mV/s. These results suggest that the oxidation and reduction of EP are primarily controlled via the diffusion process.

### 3.4. Chronoamperometric Studies

**Figure 6:**
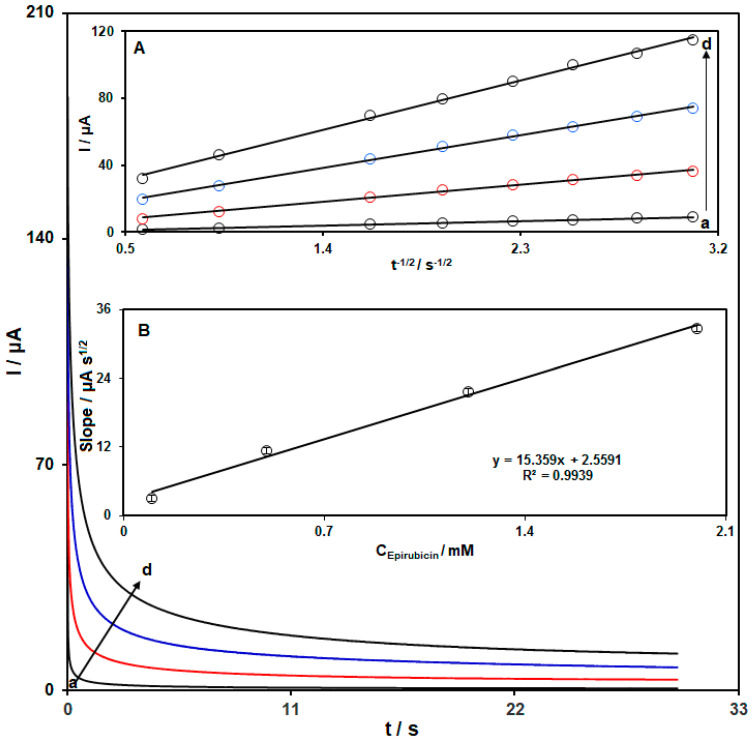
Chronoamperograms obtained at UiO-66-NH_2_/GO/SPGE in 0.1 M PBS (pH = 7.0) containing various concentrations of EP (from a to d: 0.1 mM, 0.5 mM, 1.2 mM, and 2.0 mM). Insets: (**A**) plots of Ipa versus t^−1/2^ (s^−1/2^) obtained from chronoamperograms. (**B**) Plot of slopes of Ipa vs. t^−1/2^ (µA·s^−1/2^) against EP concentrations.

Chronoamperometric measurement of EP at UiO-66-NH_2_/GO/SPGE was investigated by maintaining the electrode potential at 670 mV for various concentrations of EP in 0.1 M PBS (pH = 7.0) (Figure 6). The Cottrell equation (equation 2): (I = nFAD^1/2^Cπ^−1/2^t^1/2^) I = current response; n = number of transferred electron; F = Faraday constant (96,485 C/mol), A = geometric surface of the electrode; C = bulk concentration of EP; D = diffusion coefficient; t = time) allows for determining the diffusion coefficient (D) of an electro-active material, and its corresponding current. For the determination of the best fit for various concentrations of EP, experimental plots of Ipa versus the inverse square root of time (t^−1/2^) were utilized (Figure 6A). The slopes of the resulting straight lines obtained from the experimental plots were then plotted against the EP concentrations (Figure 6B). The D was estimated using the resulting slope obtained from the plot and the Cottrell equation. After performing the calculations, the mean value of D was determined to be 2.0 × 10^−5^ cm^2^/s.

### 3.5. The Calibration Curve

**Figure 7:**
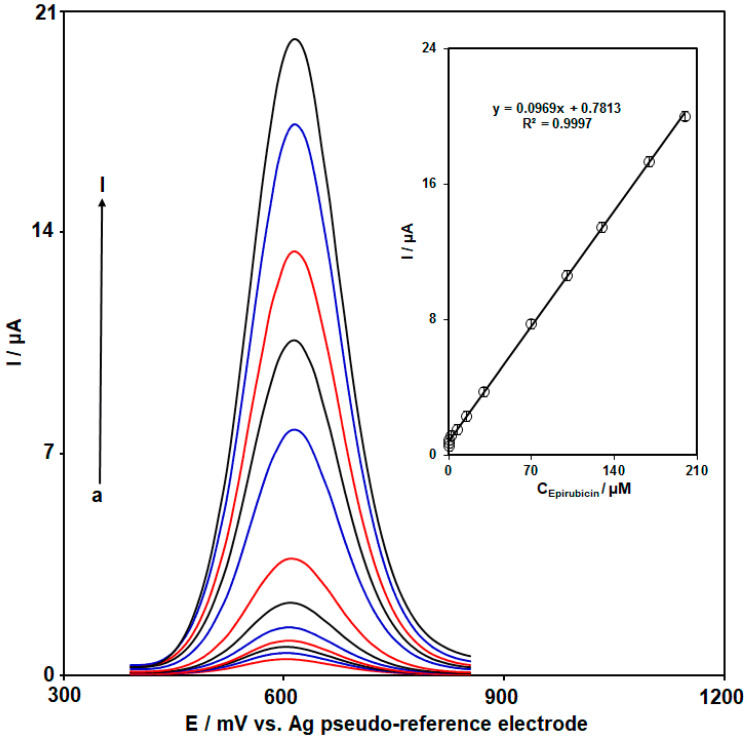
DPVs obtained at UiO-66-NH_2_/GO/SPGE in 0.1 M PBS (pH = 7.0) containing various concentrations of EP (from a to l: 0.008 µM, 0.07 µM, 0.5 µM, 2.5 µM, 7.5 µM, 15.0 µM, 30.0 µM, 70.0 µM, 100.0 µM, 130.0 µM, 170.0 µM, and 200.0 µM). Inset: The plot of the Ipa as a function of EP concentration.

DPV has been employed for quantitative measurements of EP due to its higher sensitivity compared to CV. So, DPV was employed to obtain information about the electrochemical response of the UiO-66-NH_2_/GO/SPGE sensor towards EP. Figure 7 illustrates the DPVs obtained at the proposed sensor for different concentrations of EP in 0.1 M PBS at a pH of 7.0. Upon optimizing the experimental conditions, it was observed that the oxidation peak currents exhibited a linear relationship with the concentrations of EP in the range of 0.008 to 200.0 µM. The linear regression equation describing this relationship was determined to be Ipa (µA) = 0.0969C_EP_ (µM) + 0. 7813, with an excellent coefficient of determination (R^2^) value of 0.9997. As the concentration of EP was further increased beyond the range of 0.008 µM to 200.0 µM, the peak current demonstrated a gradual increase. The detection limit for EP was calculated to be 0.003 µM, determined at a signal-to-noise ratio of 3.0. The comparison of the performance of the developed UiO-66-NH_2_/GO/SPGE sensor in the present work with previously reported sensors for voltammetric determination of EP is given in Table 1. The comparison of analytical parameters such as LOD and linear range of UiO-66-NH_2_/GO/SPGE sensor in the present work with some reported electrochemical sensors for determination of EP showed that the performance of the prepared sensor in this work is better or comparable than other sensors.

### 3.6. Simultaneous Determination of EP and TP

**Figure 8:**
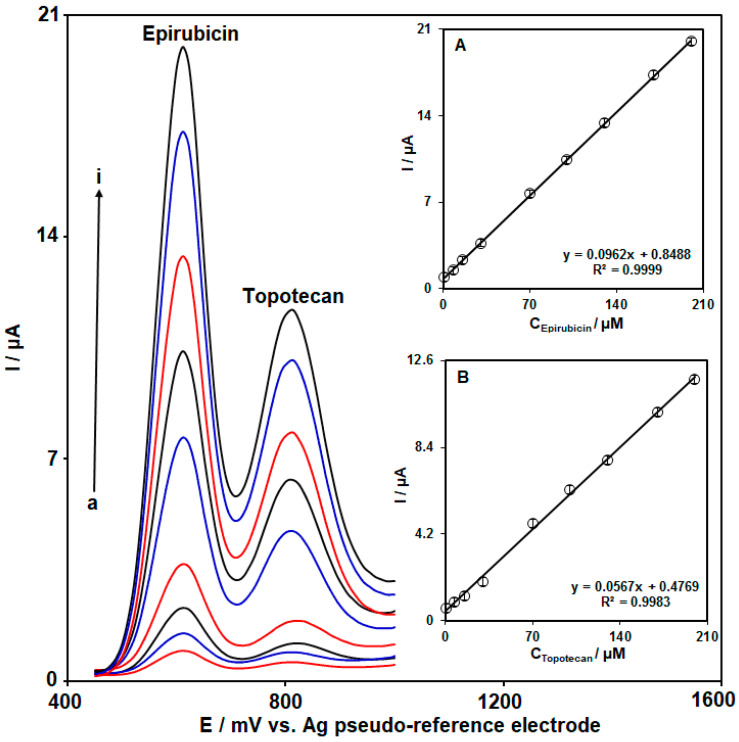
DPVs obtained at UiO-66-NH_2_/GO/SPGE in 0.1 M PBS (pH = 7.0) containing various concentrations of EP and TP (from a to i: 0.5 µM + 0.5 µM, 7.5 µM + 7.5 µM, 15.0 µM + 15.0 µM, 30.0 µM + 30.0 µM, 70.0 µM + 70.0 µM, 100.0 µM + 100.0 µM, 130.0 µM + 130.0 µM, 170.0 µM + 170.0 µM, and 200.0 µM + 200.0 µM). Insets A: The plot of the Ipa as a function of EP concentration. Inset B: The plot of the Ipa as a function of TP concentration.

To investigate the potential of UiO-66-NH_2_/SPGE for simultaneous detection of EP and TP, the study measured the DPV peak current responses of these substances while varying their concentrations in a mixture (Figure 8). Based on Figure 8, two clearly separated peaks are observed at potentials of 610 mV and 810 mV, indicating the oxidation of EP and TP, respectively. As depicted in Figure 8, the utilization of the UiO-66-NH_2_/GO/SPGE sensor can result in the differentiation of the anodic peaks of the two specified compounds, allowing for the simultaneous determination of EP and TP. The insets A and B of Figure 8 show the calibration curves for EP and TP, respectively. The data in Figure 8 shows that the peak currents for the oxidants of EP and TP rise in direct proportion to their concentrations. It is considerable that the sensitivities of the UiO-66-NH_2_/GO/SPGE to EP in the absence (0.0969 µA/µM) and presence of TP (0.0962 µA/µM) are almost close, suggesting that the oxidation processes of EP and TP at UiO-66-NH_2_/GO/SPGE occur independently. As a result, it is possible to measure two analytes at the same time or separately without any disruption.

### 3.7. Investigating the Stability, Repeatability, and Reproducibility Features of UiO-66-NH_2_/GO/SPGE Sensor

The good repeatability, stability, and reproducibility are regarded as the excellent features of electrochemical sensors and biosensors with high efficiency. Therefore, the evaluation of these features of the prepared sensor in the present work is very important.

In order to evaluate the stability property of the prepared UiO-66-NH_2_/GO/SPGE sensor, the DPV responses of this sensor for determination of 30.0 µM EP were monitored every 5 days for 15 days. After 15 days, the prepared sensor maintained a good level of stability toward EP determination (the DPV response retained 97.1% value of its initial value after this time).

The repeatability of modified SPGE towards 30.0 µM EP was also confirmed by recording the 12 repeated measurements of DPV responses from the same sensor. After these measurements, there was no significant variation in the DPV response of the UiO-66-NH_2_/GO/SPGE sensor (the DPV response decreased by less than 4.5%).

Furthermore, the reproducibility of UiO-66-NH_2_/GO nanocomposite-modified SPGE towards EP was investigated by recording the DPV responses of five electrodes in the same conditions. These electrodes showed almost identical DPV responses with an RSD value of 3.3%, demonstrating good reproducibility.

### 3.8. Selectivity Studies

To assess the selectivity of UiO-66-NH_2_/GO/SPGE sensor, the DPV measurements were conducted in 0.1 M PBS containing 25.0 µM EP in the presence of various interferants. According to the obtained results, 100-fold excess of glucose, dopamine, L-cysteine, glycine, and tryptophan; 300-fold excess of Na^+^, K^+^, Br^−^, and NO_3_^−^ showed no significant influence on the current response of EP (the signal change was less than ±5%).

### 3.9. Real Sample Analysis

To assess the practical performance of the UiO-66-NH_2_/GO/SPGE sensor, it was utilized to determine trace concentrations of EP and TP that were spiked into different real samples (EP and TP injection samples). The results are displayed in Table 2. The recovery rates of the spiked specimens were analyzed and found to range from 96.7% to 103.4%. These results indicate that the proposed method, employing the developed sensor, is suitable for practical application.

## 4. Conclusions

A simple synthesis of UiO-66-NH_2_/GO has been created and utilized for the simultaneous detection of EP and TP. The UiO-66-NH_2_/GO nanocomposite is cast onto the SPGE, which helps to enhance both electron transfer and mass transport, as well as improve electro-catalytic activity. Under optimized experimental conditions, the linear calibration curve for EP was in the linear range from 0.008 M to 200.0 M, with an LOD of 0.003 µM determined using linear regression analysis. In DPV measurements, there is an approximate 200 mV separation between the oxidation peak potentials of EP and TP at the UiO-66-NH_2_/GO/SPGE surface. Moreover, satisfactory recovery results were achieved for the detection of EP and TP in EP and TP injection samples, suggesting that the offered voltammetric technique is suitable for analysis.

## Figures and Tables

**Table 1:** Comparison of the proposed sensor with other previously reported electrochemical sensors for voltammetric quantification of EP.

Electrochemical Sensor	Electrochemical Method	Linear Range	LOD	Ref.
Fe_3_O_4_-SWCNTs/ionic liquid/CPE	Square wave voltammetry (SWV)	0.02 µM to 700 µM	0.007 µM	[4]
Pt/single-walled carbon nanotube (SWCNT)/deep eutectic solvent/carbon paste electrode (CPE)	DPV	0.001 µM to 500 µM	0.8 nM	[10]
Au@NiFe_2_O_4_/SPE	DPV	0.01 µM to 3.6 µM	0.0053 µM	[59]
CoFe_2_O_4_ nanoparticles/ionic liquid/CPE	SWV	0.04 µM to 450 µM	0.01 µM	[60]
Ag/MWCNTs/glassy carbon electrode (GCE)	SWV	0.003 µM to 0.25 µM	0.001 µM	[61]
UiO-66-NH_2_/GO/SPGE	DPV	0.008 µM to 200.0 µM	0.003 µM	This work

**Table 2:** Determination of EP and TP in real specimens using UiO-66-NH_2_/GO/SPGE sensor.

Real Sample	Spiked Concentration		Detected Concentration		Recovery		R.S.D. (n = 5)	
	EP	TP	EP	TP	EP	TP	EP	TP
Epirubicin Injection	0	0	2.9 µM	-	-	-	3.4%	-
	2.0 µM	5.0 µM	4.8 µM	5.1 µM	97.9%	102.0%	2.7%	3.2%
	4.0 µM	7.0 µM	7.0 µM	6.9 µM	101.5%	98.6%	2.1%	1.9%
	6.0 µM	9.0 µM	9.2 µM	8.8 µM	103.4%	97.8%	3.0%	2.2%
	8.0 µM	11.0 µM	10.8 µM	11.2 µM	99.1%	101.8%	2.9%	2.6%
Topotecan Injection	0	0	-	2.6 µM	-	-	-	1.9%
	5.0 µM	2.5 µM	5.1 µM	5.0 µM	102.0%	98.0%	2.4%	3.5%
	6.0 µM	3.5 µM	5.8 µM	6.3 µM	96.7%	103.3%	2.6%	2.4%
	7.0 µM	4.5 µM	7.1 µM	6.9 µM	101.4%	97.2%	3.0%	1.8%
	8.0 µM	5.5 µM	7.9 µM	8.2 µM	98.7%	101.2%	2.1%	3.7%

## Data Availability

The data presented in this study are available on request from the corresponding authors.
